# SV40 associated miRNAs are not detectable in mesotheliomas

**DOI:** 10.1038/sj.bjc.6605848

**Published:** 2010-08-17

**Authors:** G V Gee, M L Stanifer, B C Christensen, W J Atwood, D Ugolini, S Bonassi, M B Resnick, H H Nelson, C J Marsit, K T Kelsey

**Affiliations:** 1 Department of Pathology and Laboratory Medicine, Brown University, Box G-E-5, Providence, RI, 02912, USA; 2 Graduate Program in Molecular Biology, Cell Biology, and Biochemistry, Brown University, Providence, RI, USA; 3 Department of Molecular Biology, Cell Biology, and Biochemistry, Brown University, Providence, RI, USA; 4 Department of Community Health, Brown University, Providence, RI, USA; 5 Department of Oncology, Biology and Genetics, University of Genoa, Genoa, Italy; 6 Department of Epidemiology and Prevention, Units of Epidemiology and Biostatistics, National Cancer Research Institute of Genoa, Genoa, Italy; 7 Masonic Cancer Center, Division of Epidemiology, University of Minnesota, Minneapolis, MN, 55455, USA; 8 Department of Environmental Epidemiology, National Cancer Research Institute, Unit of Clinical and Molecular Epidemiology, San Raffaele Pisana, Rome, Italy; 9 Division of Epidemiology and Communicty Health, Masonic Cancer Center, University of Minnesota School of Public Health, Minneapolis, MN, USA

**Keywords:** mesothelioma, microRNA, SV40

## Abstract

**Background:**

Simian virus-40 (SV40) is a DNA tumour virus that was introduced into the human population with contaminated poliovirus vaccine, and its role in mesothelioma is widely debated. PCR based testing has been called into question, as false positives can be because of cross-reactivity with related viruses, or to laboratory contamination. The Institute of Medicine has recommended the development of more sensitive and specific tests to resolve this controversy.

**Methods:**

We have characterized highly sensitive RT–PCR based assays that are specific for *SV40-encoded* microRNAs (miRNAs), as an alternative to current testing methods.

**Results:**

Using this sensitive and specific detection method, we were unable to identify SV40 miRNA expression in human malignant pleural mesothelioma (MM) samples.

**Conclusion:**

Our work indicates that SV40 miRNAs are not likely to contribute to mesothelioma tumourogenesis, but highlights the value of this approach when compared with the relatively unspecific current testing methods.

Simian virus-40 (SV40) is a monkey polyomavirus that was first introduced into the human population in the 1950s with contaminated poliovirus vaccine, and continued until at least the early 1970s (Sweet and Hilleman, 1960). SV40 has been shown to cause tumours in hamsters and sporadically produce small quantities of infectious virus (Eddy *et al*, 1961; Sabin and Koch, 1963). The transforming roles of the early viral proteins, large T antigen and small t antigen, have been clearly established *in vitro* (Frisque *et al*, 2006; Khalili *et al*, 2008). The fact that SV40 can have cytopathic effects and can transform human cells has led to the debate as to whether this virus can cause cancer in humans.

Notably, SV40 has been implicated as a co-carcinogen in MM. Published reports have detected SV40 DNA in up to 50% of MM patients (Carbone *et al*, 1994). In 2002, a study was published by the Immunization Safety Review Committee of the United States Institute of Medicine, which explored the correlation between SV40 exposure and human cancers. The conclusion was that the evidence was inadequate to determine a causal relationship but did recommend the development of sensitive and specific serological tests for the virus (Stratton *et al*, 2002).

A recent report has demonstrated that SV40-encoded microRNAs (miRNA) are expressed in infected cells (Sullivan *et al*, 2005). The five known SV40 miRNAs are different length splice variants produced from the 5′ and 3′ arms of a precursor miRNA, derived from the late transcript, a portion generally not included in laboratory plasmids. SV40 miRNAs are distinguishable from the miRNAs encoded by the closely related human polyomaviruses, with the 5′ and 3′ mature sequences having only 50 and 77% identity to the JCV and BKV sequences (Seo *et al*, 2008). For these reasons, we concentrated on the SV40-encoded miRNAs to develop a more sensitive and reliable SV40 detection technique.

## Methods

### Viruses and cell lines

We used SVG-A cells, a subclone of SVG cells (ATCC no. CRL-8621), an SV40-transformed human astroglial cell line and SV-T2 cells (ATCC no. CCL-163.1, Manassas, VA, USA), a mouse fibroblast cell line transformed with SV40. We also used SV40-negative human embryonic kidney cells (HEK)-293 cells (ATCC no. CRL-1573), a human kidney cell line transformed with adenovirus 5 DNA. All cell lines were grown in ATCC recommended media supplemented with foetal bovine serum under standard conditions.

### Tissue ascertainment

Fresh frozen tissues were obtained from the CREST Biorepository of the National Cancer Research Institute, Genova, Italy, the Masonic Cancer Center's Tissue Procurement Facility at the University of Minnesota, the National Mesothelioma Virtual Bank, the tumour bank at Rhode Island Hospital and the National Disease Research Interchange. The samples consisted of non-malignant biopsies (*n*=28), pleural biopsies from lung adenocarcinoma patients (*n*=20) and a mixture of 94 mesothelioma tumour samples, made up of epithelioid (*n*=42), biphasic (*n*=18) sarcomatoid (*n*=10) and non-histologically defined mesothelioma patients (*n*=24). Data on patient age was not available for all samples, but for known tumours, the average patient age was 62.3 years for mesotheliomas and 75.2 years for lung adenocarcinomas and matched normal tissue.

### Infections and RNA isolation

Cells were grown to 70% conuence on coverslips and incubated with 1 × 10^5^ PFU of SV40 (wild type strain 777) per 1 × 10^6^ cells for 1 h at 37 °C. At the end of the incubation, cells were washed and fed with Eagle's minimal essential medium for up to 96 h. The media was then aspirated off and cells were detached with trypsin-EDTA for 5 min at 37^o^C. Cells were pelleted and washed two times with cold PBS. Infections were performed in triplicate and three samples of 1 × 10^6^ cells were harvested and transferred to a clean tube and pelleted. RNA extractions were performed using the Ambion *mir*Vana miRNA Isolation Kit (cat no. AM1561, Applied Biosystems/Ambion, St. Austin, TX, USA) according to the manufacturer's instructions for total RNA and small RNA-enriched fractions. All live virus work was performed in a laboratory separate from RNA and PCR work.

### Quantitative PCR assays and kits

RNA Oligonucleotides were purchased from Invitrogen Corporation, Carlsbad, CA, USA and IDT (Integrated DNA Technologies, Inc., Coraville, IA, USA). All miRNA assays were purchased from (Applied Biosystems, Foster City, CA, USA). Human: RNU44 (part no. 4373384), RNU48 (part no. 4373383), RNU6B (part no. 4373381). Mouse: snoRNA202 (part no. 4380914). Viral specific miRNA assays are based on 5′ and 3′ mature miRNA sequences and are available to purchase from Applied Biosystems. Assay validations were performed on using synthetic RNA oligos diluted to 10 *μ*M in water. A total of 5 *μ*l of oligo was reverse transcribed and amplified by quantitative PCR in triplicate according to the manufacturer's directions to test the specificity of each miRNA assay. Experiments on cell lysates used 10 ng of total RNA or RNA enriched for small RNAs as template. All cDNA synthesis and quantitative PCR were performed according the manufacturers’ conditions and run on an Applied Biosystems’ 7300 Real Time PCR System. TaqMan MicroRNA Reverse Transcription Kit, 1000 reactions (part no. 4366597), High Capacity cDNA Reverse Transcription Kit with RNase Inhibitor, 200 Reactions (part no. 4374966), TaqMan Universal PCR Master Mix, No AmpErase UNG, 1-Pack (1 × 5 ml; part no. 4324018) were used.

## Results

### Assay validation

**Figure 1:**
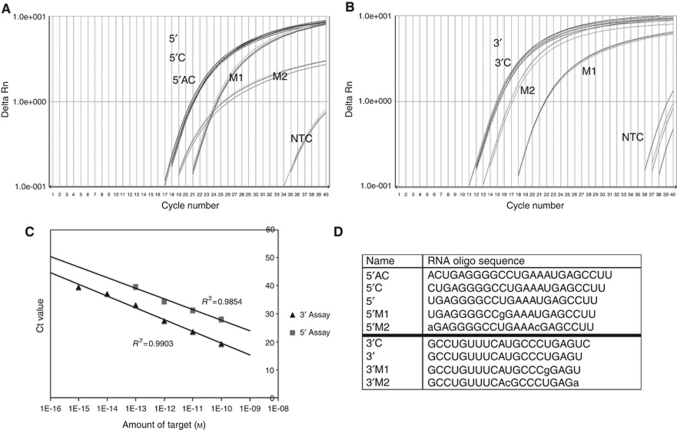
MicroRNA assay validation. Assays designed against the minimal 5′ and 3′ miRNA sequences were tested using synthetic RNA oligonucleotides. (**A**) The assay designed against the minimal 5′ miRNA sequence was tested using the 5′, 5′C and 5′AC synthetic oligos as well as 5′M1, a mismatched oligo otherwise identical to 5′ with a U → G substitution at position 10, and 5′M2, a oligo mismatched to 5′ with a U → A substitution at position 1 and a U → C substitution at position 15. (**B**) The assay designed against the minimal 3′ miRNA sequence was tested using the 3′, and 3′C synthetic oligos as well as 3′M1, a mismatched oligo otherwise identical to 3′ with a U → G substitution at position 16, and 3′M2, a mismatched oligo to 3′ with a U → C substitution at position 11 and a U → A substitution at position 20. Amplification plots reveal that neither the 5′ nor the 3′ assay can differentiate between the minimal and longer mature miRNAs but they are specific for the sequences. The experiment was repeated using assays designed against the longer 5′AC, 5′C and 3′C miRNA sequences. These yielded results similar to the assays designed against the minimal sequences (data not shown). (**C**) qRT–PCR was performed on serial dilutions of RNA oligos to determine the linearity of the assay over several orders of magnitude. (**D**) The sequences of RNA oligos used to test each assay. Mismatched nucleotides are shown in lowercase.

We used custom real-time quantification PCR assays developed by Applied Biosystems that employ a stem-loop real-time PCR primer for the specific detection of mature miRNA (Chen *et al*, 2005). This approach allows for highly specific detection of the active miRNA product, rather than an inconclusive result in the detection of the longer, unspliced, pre-miRNA. Five specific assays were developed, one for each of the three different 5′ splice variants (5′AC, 5′C and 5′) and one each for the two different 3′ splice variants (3′C and 3′). Each assay was validated using cDNA made from 10 nM dilutions of synthetic RNA oligonucleotides (Figure 1). The assays were tested for fidelity with synthetic RNA oligonucleotides containing either one or two mismatches to the minimal 5′ and 3′ sequences (Figure 1D). Neither the 5′ assay nor the 3′ assay could detect differences between the minimal miRNA and the longer splice variants, but they were specific for the sequences themselves. Each assay discriminated the mismatched oligonucleotides, although the position of the mismatches influenced the sensitivity of the assays. The single mismatch U → G had less cross-reactivity in both cases than the combined U → A and U → C mismatches. Owing to the ability of the minimal 5′ and 3′ assays to detect the longer respective miRNA splice variants, the minimal assays were used in all future experiments (Figures 1A and 1B). Dilutions of the minimal synthetic 5′ and 3′ RNA oligos were used to measure the sensitivity of the assays, which are linear over multiple orders of magnitude (Figure 1C).

### SV40 miRNAs are not expressed in transformed cells

**Figure 2:**
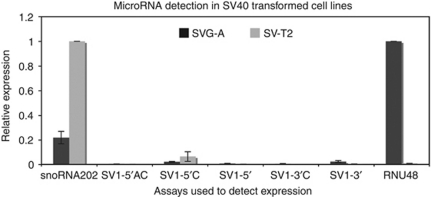
Relative background detection in SV40-transformed cell lines. Assays against the various miRNAs were tested for fidelity using the mouse SV40-transformed cell line, SV-T2, and the human SV40-transformed cell line, SVG-A. Cell lysates enriched for small RNAs were reverse transcribed using each specific assay. Quantitative PCR was performed to measure the levels of each SV40 miRNA in relation to the mouse control small RNA, snoRNA202, and the human control small RNA, RNU48. Error bars represent the s.e. of two experiments for SV-T2 cells or three experiments for SVG-A cells. The relative quantification of miRNA was performed according to the 2^−ΔΔC_t_^ method, comparing product amplified from mouse cells relative to snoRNA202, and product amplified from human cells relative to RNU48.

We next used SV40-transformed mouse (SV-T2) and human (SVG-A) cell lines to test the sensitivity of all five assays compared with the species-specific small RNAs, snoRNA202 (mouse) and RNU48 (human). These cells contain at least one copy of the entire genome but do not express the late transcript and therefore should not express the mature miRNAs (Botchan *et al*, 1976; Major and Matsumura, 1984). We compared the background detection of each assay in small RNA-enriched lysates of both cell types and found that both the 5′ and 3′ assays detected <1% of the relative control (Figure 2). The minimal assays had the least cross-reactivity in transformed cell lines with the 3′ and 5′ assays being specific to 100 fM and 300 fM, respectively.

### SV40 miRNA expression in infected human cells

**Figure 3:**
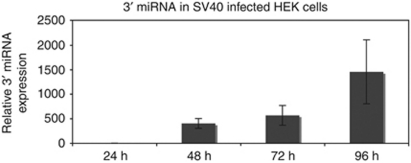
3′ MicroRNA detected in SV40-infected HEK cells over time. HEK cells were infected with SV40 in a laboratory separate from the site of RNA preparation and PCR. Infections were performed with mock controls in a blinded manner. At each time point, three samples of 1 × 10^6^ cells were harvested and total RNA was isolated. Fractions enriched for small RNAs were reverse transcribed using the 3′, 5′ and RNU48 assays. Quantitative PCR was performed to measure the levels of miRNA relative to the human control small RNA, RNU48. The relative quantification of miRNA was performed according to the 2^−ΔΔC_t_^ method, comparing product amplified from SV40-infected human cells relative to RNU48. Once it was determined that mock controls and 5′ assays amplified no product in infected HEK cells (data not shown), qPCR was repeated on HEK cells at the four time points using the 3′ and RNU48 assays on the same plate for accurate relative quantification. Error bars represent s.e. across triplicate infections.

We chose the minimal 5′ and 3′ assays to detect miRNA in actively infected cells following SV40 infection of human embryonic kidney cells HEK and measured the expression over time. We found that the 3′ miRNA was first detected 48 h after infection. The level continued to increase over 96 h of the initial round of infection in accordance with the known timeline of late transcription (Figure 3). In these cells, the 5′ transcript was undetectable during the entire 96-h time course (data not shown).

### SV40 miRNA not detected in human mesothelioma biopsies

**Figure 4:**
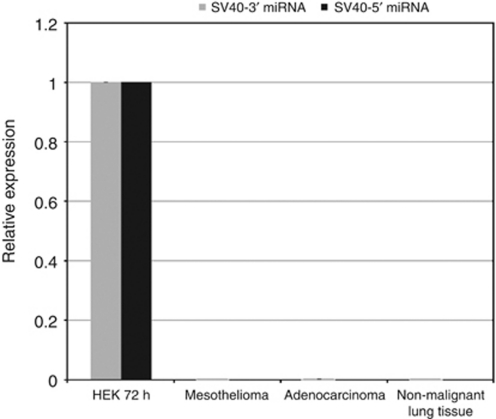
Quantitative RT–PCR of SV40 microRNA. Quantitative RT–PCR was performed on 94 human malignant mesothelioma biopsies, 28 nonmalignant biopsies and 20 lung adenocarcinomas. Quantitative PCR was performed to measure the levels of miRNA relative to the human control small RNA, RNU48. The relative quantification of miRNA was performed according to the 2^−ΔΔC_t_^ method and measurements were made relative to RNA isolated from HEK cells 72 h after SV40 infection.

We used the minimal 5′ and 3′ assays to detect the expression of SV40-encoded miRNAs in 94 human malignant mesothelioma tumour biopsies as well as in 20 lung adenocarcinoma cancerous and 28 non-malignant control biopsies (Figure 4). Despite the high sensitivity of these assays, we were unable to find any evidence of SV40 miRNA expression in human tissues. To ensure that the lack of SV40 miRNA expression in mesotheliomas was not because of technical problems isolating miRNA from these tissue samples, or to a general miRNA processing defect of this tumour type, we used quantitative PCR to detect three separate host-encoded miRNAs in mesotheliomas compared with non-malignant lung tissue (Supplementary Figure 1).

## Conclusion

We characterized sensitive and specific quantitative PCR assays that are based on the expression of the mature SV40-encoded miRNAs. We showed that, although the assays cannot distinguish the minimal 5′ and 3′ miRNAs from the longer splice variants, the assays are highly sensitive to sequence. Single mismatches in the detected sequence result in decreased amplification. We used synthetic oligonucleotides to make standard curves and show that both minimal 5′ and 3′ assays are sensitive over many orders of magnitude. We also used SV40-transformed and non-SV40-transformed cell lines to characterize the levels and timeline of miRNA expression following infection with the virus.

Our results indicated that these assays were both sensitive and specific enough to meet the standard for serological SV40 tests recommended by the Institute of Medicine. We have tested these assays on 94 different human MM samples from multiple tissue banks. Using the 5′ and 3′ assays, we were unable to detect SV40 miRNAs in any human tissue samples. The initial paper describing these miRNAs suggested that they may function to downregulate SV40 early protein expression and allow the virus to escape immune clearance (Sullivan *et al*, 2005). This led us to postulate that these miRNAs may be detectable in mesotheliomas and could provide a useful target to develop a more sensitive test for the virus. From our work, we have concluded that mature SV40 miRNA are not detected in human tissue samples, indicating that they are not likely to contribute to mesothelioma tumourigenesis. This can imply that the virus itself does not contribute to the development of mesothelioma. Another interpretation is that, because only the early SV40 genes are necessary to transform cells, only these genes are expected to be expressed in mesotheliomas (Carbone *et al.*, 2008). The lack of false-positive results in an array of cancerous and non-cancerous human tissue shows that these assays are less vulnerable to laboratory contamination and cross-reactivity. The consistent lack of detection of SV40 miRNAs in human malignant mesotheliomas is indicative of a lack of association of miRNA-producing infectious SV40 with this tumour type. We cannot, however, completely exclude the association of human mesothelioma with a transforming form of SV40.
