# Supplementary material for: SV40 associated miRNAs are not detectable in mesotheliomas
Source: Br J Cancer. 2010 Aug 17;103(6):885–8. doi: 10.1038/sj.bjc.6605848 (PMC2966630; doi:10.1038/sj.bjc.6605848)
Supplement: Supplementary Figure 1 [file 6605848x1.doc]

**Supplementary figure 1: Detection of SV40-Unrelated miRNA in Tissue Specimens.** To determine whether we are could detect miRNAs processed and expressed in mesotheliomas, we compared the expression of three host-encoded miRNAs between ten mesothelioma biopsies and ten non-malignant lung tissue samples. Quantitative PCR was performed to measure the levels of miRNA relative to the human control small RNA, RNU48. The relative quantification of miRNA was performed according to the **2^-DDCt^** method. Assays were purchased from Applied Biosystems. Human RNU48 (part # 4373383), hsa-miR-99b (part # 4427975), hsa-miR-130a (part # 4427975) hsa-miR-222 (part # 4427975).
